# Investigation of serum biomarkers in rheumatoid and psoriatic arthritis patients for disease-specific signatures

**DOI:** 10.1186/s13075-025-03608-6

**Published:** 2025-07-10

**Authors:** James D Veale, Áine Gorman, Douglas J Veale, Ursula Fearon, Carl Orr, Viviana Marzaioli

**Affiliations:** 1 Molecular Rheumatology, School of Medicine, Trinity Biomedical Sciences Institute, Trinity College Dublin, Dublin, Ireland; 2 Rheumatology EULAR Centre of Excellence, Centre for Arthritis & Rheumatic Diseases, St Vincent’s University Hospital, University College Dublin, Dublin, Ireland

**Keywords:** Rheumatoid arthritis, Psoriatic arthritis, Serum biomarkers, Inflammation, Disease pathotypes.

## Abstract

**Background:**

Rheumatoid arthritis (RA) and Psoriatic arthritis (PsA) are systemic auto-immune diseases of unknown aetiology that lead to systemic inflammation and synovial joint destruction. Identification of specific serum proteins that selectively regulate these diseases, or which precede disease development could have great potential as disease biomarkers and predictors.

**Methods:**

Serum levels of C-reactive protein (CRP), sICAM-1, sVCAM-1, Serum amyloid A (SAA), Matrix metalloproteinases (MMPs 1, 3 and 9) and metabolic markers: Active Glucose-dependent Insulinotropic polypeptide (GIP), active Glucagon-like peptide-1 (GLP-1), C-Peptide, Glucagon, Insulin, Leptin, Pancreatic Polypeptide (PP) were measured by Meso Scale Discovery (MSD) multiplex analysis assay.

**Results:**

Serum levels of sICAM-1, MMP1, MMP3, PP, c-Peptide, CRP and SAA were specifically upregulated in RA, but not in PsA disease, displaying high sensitivity (ROC curves). In the early phase of the disease, these markers may be suitable for discriminating RA from PsA patients. Differences in sex, BMI, and disease activity were observed. This is the first study which directly compare serum metabolic markers between diseases and identifies specific disease signatures between RA and PsA. In addition, this study identified that CRP, SAA, GLP-1, GIP-1, Leptin and PP serum protein precede disease onset, as they are already altered in the serum of ‘individuals at risk’ of developing RA. Of these, CRP, SAA, Leptin and PP might predict IAR conversion to RA^+^, thus making them suitable candidates for disease prediction.

**Conclusions:**

Altogether, this study identifies selective serum markers associated with RA and PsA, which are pathotype-specific and are predictors of RA disease onset.

**Supplementary Information:**

The online version contains supplementary material available at 10.1186/s13075-025-03608-6.

## Background

Rheumatoid arthritis (RA) and Psoriatic arthritis (PsA) are two autoimmune systemic diseases, affecting ~ 1% of the population worldwide, that lead to synovial joint inflammation, physical disability, and increased mortality [1–3]. Treating RA and PsA patients early is imperative to reducing disease progression and improving long-term outcomes, therefore latest research is aimed at the identification of biomarkers in serum and blood, which could predict disease onset and progression. Serologic studies are often used in RA clinics to determine the presence of autoantibodies, specifically anti–citrullinated protein antibodies (ACPAs) and rheumatic factor (RF) [4–7], which have been shown to be detectable months to years prior to the onset of clinically identifiable disease, thus serving as current biomarkers to identify ‘individuals at risk’ (IAR) who may subsequently develop RA [8, 9]. These markers, however, do not account for seronegative RA patients and PsA patients, who normally lack these autoantibodies. Currently, CRP and SAA are often measured in RA and PsA patients to evaluate inflammatory status and have become the gold standard in monitoring a wide spectrum of inflammatory diseases [10]. In addition, soluble ICAM-1 and VCAM-1 have been shown to be increased in the serum of RA patients and correlate with disease activity [11]. New technologies, such as multi proteomic approaches, have been utilised in the development of protein marker panels that are specific for RA vs. PsA [12]. However, further studies are required to validate biomarkers that distinguish specific patient groups, and importantly needed for identification of novel biomarkers which could predict RA onset and progression, as well as the insurgence of co-morbidity, including metabolic dysfunction.

This study identifies selective vascular, metabolic and MMPs markers which are differentially expressed in the serum of RA and PsA patients, compared to healthy controls (HC), and are mostly specific for the RA pathotype. In addition, some of these markers preceded disease onset, as selective proteins were already altered in the serum of IAR patients compared to HC. ROC curves and correlation studies confirmed that the measurement of vascular, metabolic and MMP markers together, could aid the identification of disease-specific profiles, onset, and progression.

## Methods

### Patient demographics

Blood was collected from healthy controls (HC *n* = 15), individuals at risk (IAR *n* = 36), RA (*n* = 74) and PsA (*n* = 97) patients. \Demographics, including age, sex, Disease Activity Score 28 (DAS-28-CRP), Body Max Index (BMI) and treatments are found in Table 1. RA were defined by the revised ACR 2010 criteria and PsA classified according to the CASPAR criteria. All individuals gave full written consent, and ethics were approved by St. Vincent’s University Hospital research ethics committee, Dublin, Ireland.

**Table 1:**
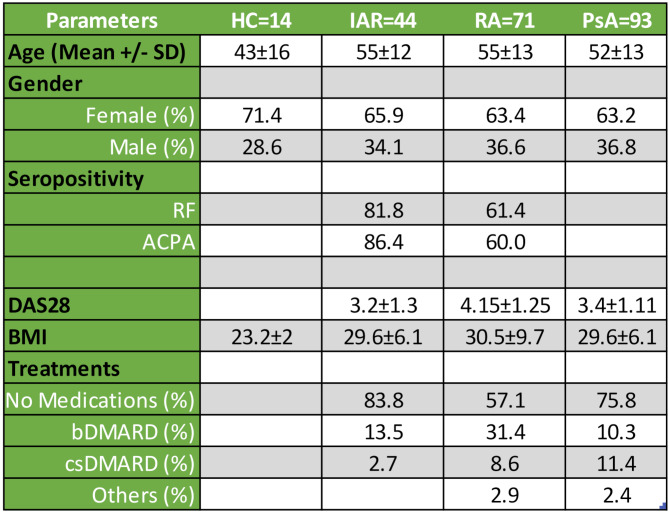
Patient demographics. Age

### Serum collection

Bloods were collected and centrifuged for 5 min at 1800 rpm to collect serum. Serums were aliquoted and stored at -80 °C until usage.

### Multiplex analysis

Serum levels of C-reactive protein (CRP), sICAM-1, sVCAM-1, Serum amyloid A (SAA), Matrix metalloproteinases (MMPs 1, 3 and 9) and metabolic markers: Active Glucose-dependent Insulinotropic polypeptide (GIP), active Glucagon-like peptide-1 (GLP-1), C-Peptide, Glucagon, Insulin, Leptin, Pancreatic Polypeptide (PP) were measured using Meso Scale Discovery (MSD) multiplex analysis assay (Meso Scale Diagnostics, USA) according to the manufacturer’s protocol.

### Statistical analysis

Data were analysed with GraphPad Prism 10 software. Differences between groups were analysed by the Non-parametric One-way analysis of variance (ANOVA), Kruskal-Wallis test, with Dunn’s post-hoc test (*) or non-parametric Mann-Whitney t-test (#) and */^#^*p* < 0.05, **/^##^*p* < 0.01, ***/^###^*p* < 0 0.001, ****/^####^ *p* < 0.0001 values were considered as significant. Multivariable analyses were performed between age, BMI, gender and DAS28-CRP, and represented as Bubble plot. In addition, multivariable correlation matrices were analysed with non-parametric Spearman correlations, with 95% Confidence Interval. Principal component analysis (PCA) was performed with a standardized scale, and loading plots generated. Receiver operator characteristic (ROC) curves were generated with the Wilson/Brown methods and area under the curve were generated. The specific test used can be found detailed in corresponding figure legends.

## Results

### Vascular, metabolic and MMP serum markers are differentially increased in RA and PsA patients

Multiplex analysis was performed to analyse multiple inflammatory and metabolic markers in the serum of RA and PsA patients, using healthy subjects (HC) as controls.

**Fig. 1:**
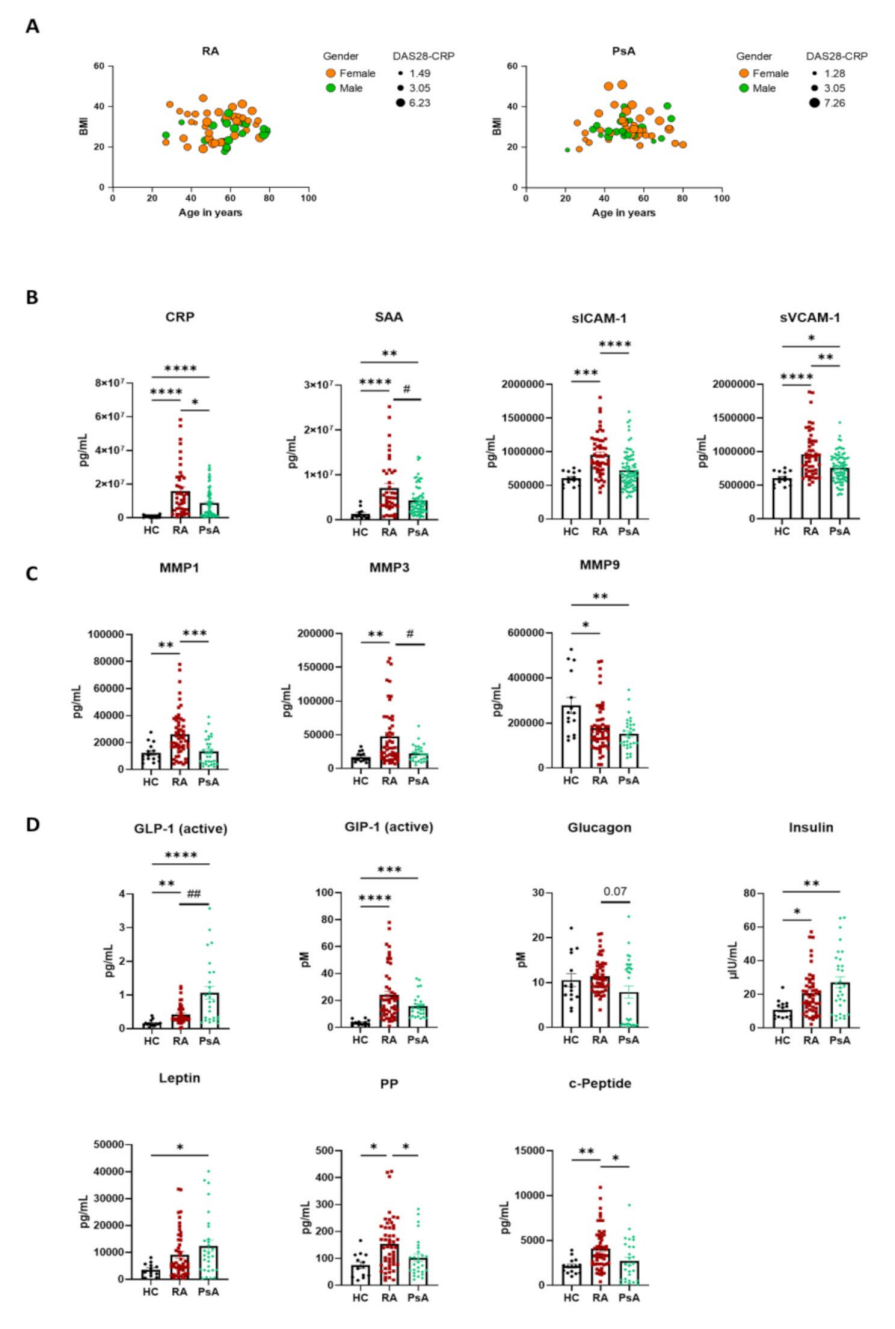
Vascular, metabolic and MMPs serum markers are differentially increased in RA and PsA patients. Serum from HC (*n* = 15), RA (*n* = 74) and PsA (*n* = 97) patients were collected, and MSD multiplex analysis was performed. **(A)** Multivariable analysis was performed and represented as a Bubble plot. Age in year (x axes), BMI (Y axes), sex as bubble colour (female orange and male green), and DAS28-CRP as bubble size (area) **(B)** vascular markers **(C)** MMPs, and **(D)** metabolic markers. Data are presented as Mean ± SEM and statistical differences among groups were obtained with non-parametric One-way ANOVA analysis (Kruskal-Wallis test with Dunn’s post-hoc test). *p < 0.05, ** *p* < 0.01, *** *p* < 0.001, **** *p* < 0001. Due the high heterogenicity of the samples, selective pairs were further analysed with Mann–Whitney t-test. ^#^*p* < 0.05, ^##^ *p* < 0.01, ^###^ *p* < 0.001, ^####^ *p* < 0001

To investigate whether our cohort was homogeneous and the impact of sex, BMI, age and DAS28-CRP, we performed multivariate analysis (Fig. 1A). Interesting, both RA and PsA clustered for the four variables in patients between 40 and 60 years of age, and BMI between 20 and 40. PsA females with a higher DAS28-CRP tended to have a higher BMI within that age frame.

A strong increase in inflammatory markers CRP and SAA was observed in RA and PsA patients when compared to HC serum (Fig. 1B *p* < 0.0001), in agreement with previous studies [13, 14]. In addition, we observed that both CRP and SAA values were higher in RA vs. PsA patients (*p* < 0.05), which is also consistent with previous observations [1, 15, 16]. Interestingly, we also observed an increase in soluble ICAM and VCAM proteins in RA vs. HC (*p* < 0.001 and *p* < 0.0001, Fig. 1B), both of which are known to be associated with disease activity [11]. Interestingly, while sVCAM-1 was increased in PsA patients compared to HC (*p* < 0.05), we did not observe a significant increase in sICAM-1 in PsA vs. HC (Fig. 1B). Both sICAM-1 and sVCAM-1 concentrations were significantly higher in RA vs. PsA (*p* < 0.0001 and *p* < 0.01, respectively).

Serum MMPs have been shown to be associated with increased inflammation and progression of joint damage in early RA [17, 18]. In this study, we observed a significant increase in MMP1 and MMP3 (*p* < 0.01) in the serum of RA patients compared to HCs; in contrast, a significant decrease in serum MMP9 was observed (*p* < 0.05) (Fig. 1C). Both MMP1 and MMP3 levels were unaltered in PsA vs. HC, showing significant differences when compared to RA (*p* < 0.001 and *p* < 0.05), while MMP9 was significantly decreased when compared to HC (*p* < 0.01).

RA and PsA are multifeatured diseases which is often associated with different co-morbidities, including obesity, cardiovascular disease and insulin resistance [19, 20], therefore we evaluated whether metabolic markers were altered in the serum of RA and PsA patients. Interestingly, we observed that active GLP-1 (*p* < 0.01), GIP-1 (*p* < 0.0001), Insulin (*p* < 0.05), PP (*p* < 0.05) and c-Peptide (*p* < 0.01) were all significantly increased in the serum of RA patients, when compared to HC (Fig. 1D). A trending increase in Leptin was also observed, however this did not reach statistical significance. Interestingly, GLP-1 levels were increased in PsA patients, at a higher level, when compared to both HC (*p* < 0.0001) and RA (*p* < 0.01). GIP-1, Insulin and Leptin were increased in PsA patient’s vs. HC (*p* < 0.001, *p* < 0.01 and *p* < 0.05). In addition, both PP and c-Peptide were unaltered in PsA vs. HC and showed significant differences with respect to RA patients (*p* < 0.05).

The mean age for RA and PsA patients is very comparable (Table 1), with the HC cohort presenting a slightly younger cohort. To investigate whether age could be a confounder for the observed serum marker regulations, all markers were correlated with age. In Supplementary Table 1, we observe that in HC and PsA, no marker expression correlated with age, while in RA, only GLP-1 correlated with age (*p* < 0.05). This suggests that the serum marker regulation observed in HC vs. RA vs. PsA, are not age dependent.

Altogether, these data suggest that circulatory serum markers are differentially regulated in RA vs. PsA patients.

We next evaluated whether these markers were correlating with disease activity, using a DAS28-CRP threshold of 3.2, to separate patients with active high disease activity, vs. lower disease activity, as per EULAR (European League Against Rheumatism) guidelines [21]. We observed that CRP, MMP1, MMP3, Leptin and PP had a trending increase in the high DAS28 (> 3.2) RA cohort vs. the low DAS28 RA cohort (< 3.2), however they did not reach significance (Supplementary Fig. 1A). In PsA, trending increases were observed for MMP9 (*p* = 0.07), Leptin (*p* = 0.06) and sICAM-1 (*p* = 0.09), in the high DAS28 vs. the low DAS28 groups.

The incidence for RA in women is greater than that observed in men, while PsA affects sexes equally, which a trending increase in men [22–24], overall however, women have a poorer prognosis in terms of long-term pain and disease activity [25, 26]. In our RA cohort, we observed that selective markers were differentially expressed among female and male patients, with sVCAM-1 (*p* = 0.06), MMP3 (*p* < 0.01) and PP (*p* < 0.05) being higher in male vs. female, and Leptin (*p* < 0.05) being higher in female vs. male (Supplementary Fig. 1B). In PsA, sICAM-1 (*p* < 0.01), sVCAM-1 (*p* = 0.08) and Insulin (*p* < 0.05) were higher in males, while Leptin (*p* < 0.01) was higher in the female cohort.

Recent studies have suggested obesity should be considered a confounding factor for RA and PsA disease severity and progression [27, 28], therefore, we stratified patients into high BMI (> 25) and low BMI (< 25) (Supplementary Fig. 1C), and observed that selective metabolic markers were higher in the RA BMI high group, including Leptin (*p* < 0.01) and c-Peptide (*p* = 0.08). In contrast, SAA (*p* < 0.05) was decreased in the high BMI group. In PsA, an increase was observed for CRP (*p* = 0.07) and GLP-1 (*p* < 0.05) high BMI group. All the other markers were unaltered between the two groups.

RA patients can be divided into two main subgroups based on the presence or absence of autoantibodies [1, 29], therefore we stratified the RA patients into seropositive (RA^+^) vs. seronegative patients (RA^−^) (Supplementary Fig. 2A), based on the presence of RF and/or ACPA. We observed that seropositive RA patients displayed increased serum markers, including CRP (*p* < 0.05), SAA (*p* = 0.05), sICAM (*p* < 0.05), sVCAM (*p* = 0.07), and GLP-1 (*p* = 0.07) GIP-1. To account for the absence of autoantibodies in PsA, we compared the above markers between RA^−^ and PsA. Interestingly, CRP was still found to be higher in RA^−^ vs. PsA (*p* < 0.05), with a trending decrease observed for sICAM-1, sVCAM-1, GLP1 and GIP-1 active, although not reaching significancy (Supplementary Fig. 2B). This might suggest that the differences between RA and PsA are not only due to the lack of autoantibodies in the latter, but might be ascribed to more complex molecular mechanisms, although bigger cohorts should be used to confirm this.

Altogether, these data suggest that the vascular, metabolic and MMP serum markers are selectively altered in specific patient subgroups in RA and PsA.

### Serum markers differentially correlate in disease pathotypes

**Fig. 2:**
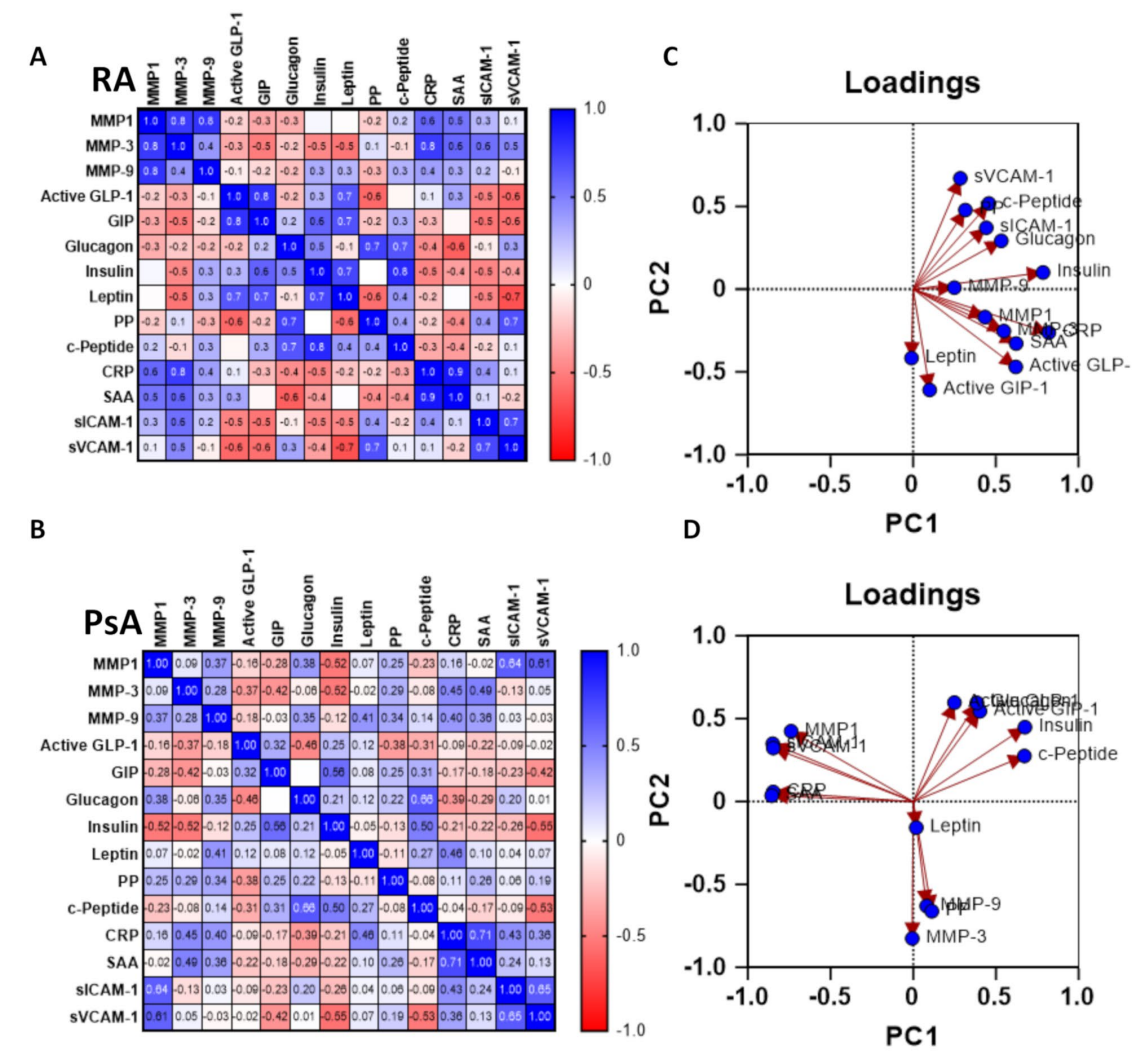
Selective serum markers are differentially correlated and clustered in RA and PsA patients. Serum from HC (*n* = 15), RA (*n* = 74) and PsA (*n* = 97) patients were collected, and MSD multiplex analysis was performed. Non-parametric Spearman correlation matrixes were obtained and heatmaps created for **(A)** RA and **(B)** PsA patients. p-values associated with the correlation matrix can be found in the Supplementary Fig. 3. PCA analysis for **(C)** RA and **(D)** PsA patients. Standardized analysis was used for the PCA analysis and loadings for PC1 and PC2 are shown

Correlation matrix and PCA analysis were performed to evaluate the relationship rate among all serum markers analysed, and to identify a predictive panel of disease-specific markers (Fig. 2). When comparing correlation matrixes between RA (Fig. 2A) and PsA (Fig. 2B), we clearly observe a different correlation distribution, which a higher number of markers correlated in RA vs. PsA (in blue shades). The analysis of the correlation matrix with non-parametric Spearman correlation, confirmed this observation (Supplementary Fig. 3). For example, MMP1 in RA was significantly correlating with MMP3, MMP9, Insulin, c-Peptide, CRP, SAA and s-ICAM-1, while in PsA it only correlated with MMP9, s-ICAM-1 and s-VCAM-1, with a trending correlation with Glucagon.

Principal component analysis (PCA) analysis revealed two defined clusters in RA (Fig. 2C), with two sets of clusters correlating together. In contrast, three clusters were identified in PsA. Cluster 1 in RA included sVCAM-1, c-Peptide, sICAM-1, Glucagon, PP, MMP9 and Insulin, while Cluster 2 included MMP1, MMP3, CRP, SAA, GLP-1, GIP-1 and Leptin, thus suggesting the markers within the two clusters might be expressed together in patients. Interestingly, in PsA the markers correlated differently in three Clusters, with Cluster 1 including GLP-1, GIP-1, Glucagon, Insulin and c-Peptide; Cluster 2 including Leptin, MMP9, PP and MMP3, and Cluster 3 including MMP1, sICAM-1, sVCAM-1, CRP and SAA (Fig. 2D). All together, these data suggest that in RA and PsA serum markers correlate and cluster differently, thus confirming significant differences between the two diseases.

### Selective serum marker sensitivity makes them great candidates as RA biomarkers

**Fig. 3:**
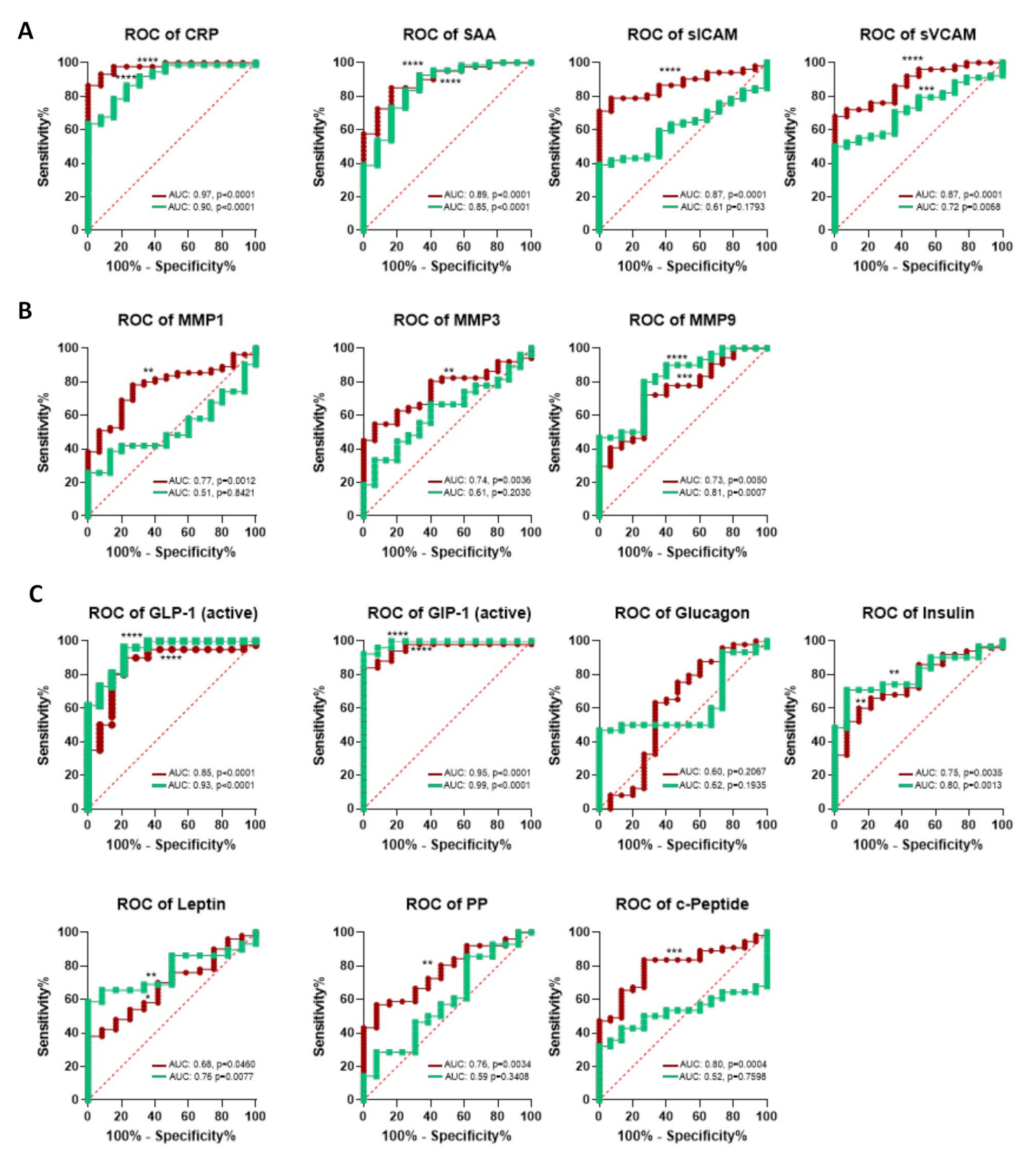
Specificity and sensitivity of serum markers in RA and PsA patients. Receiver operating characteristic (ROC) curves for RA vs. HC (in red) and PsA vs. HC (in red) with Wilson/Brown method and CI of 95%. AUC under the curves and p value are expressed for **(A)** vascular markers **(B)** MMPs, and **(C)** metabolic markers. *p < 0.05, ** *p* < 0.01, *** *p* < 0.001, **** *p* < 0001

In order to evaluate the diagnostic value of the evaluated serum markers in RA and PsA, we performed receiver operator characteristic (ROC) curve analysis, which allow measurement of the sensitivity and specificity of each marker in RA vs. PsA, when compared to HC. In RA, all markers analysed resulted in a significant ROC curve (Fig. 3, in red), except for Glucagon which showed no significant sensitivity. CRP, SAA, sICAM-1, sVCAM-1, MMP9, GLP-1, GIP-1 and c-Peptide showed the highest sensitivity. In PsA, only CRP, SAA, sVCAM-1, MMP9, GLP-1, GIP-1, Insulin and Leptin demonstrated significant sensitivity (Fig. 3 in green).

Interestingly, CRP, SAA, GLP-1 and GIP-1 fully overlapped between RA and PsA, thus making them strong candidate markers for both diseases, whereas sICAM-1, MMP1, MMP3, PP and c-Peptide were exclusively significant in RA, thus suggesting that they may be more unique as RA biomarkers.

### Selective inflammatory, metabolic mediators and MMPs precede disease onset

**Fig. 4:**
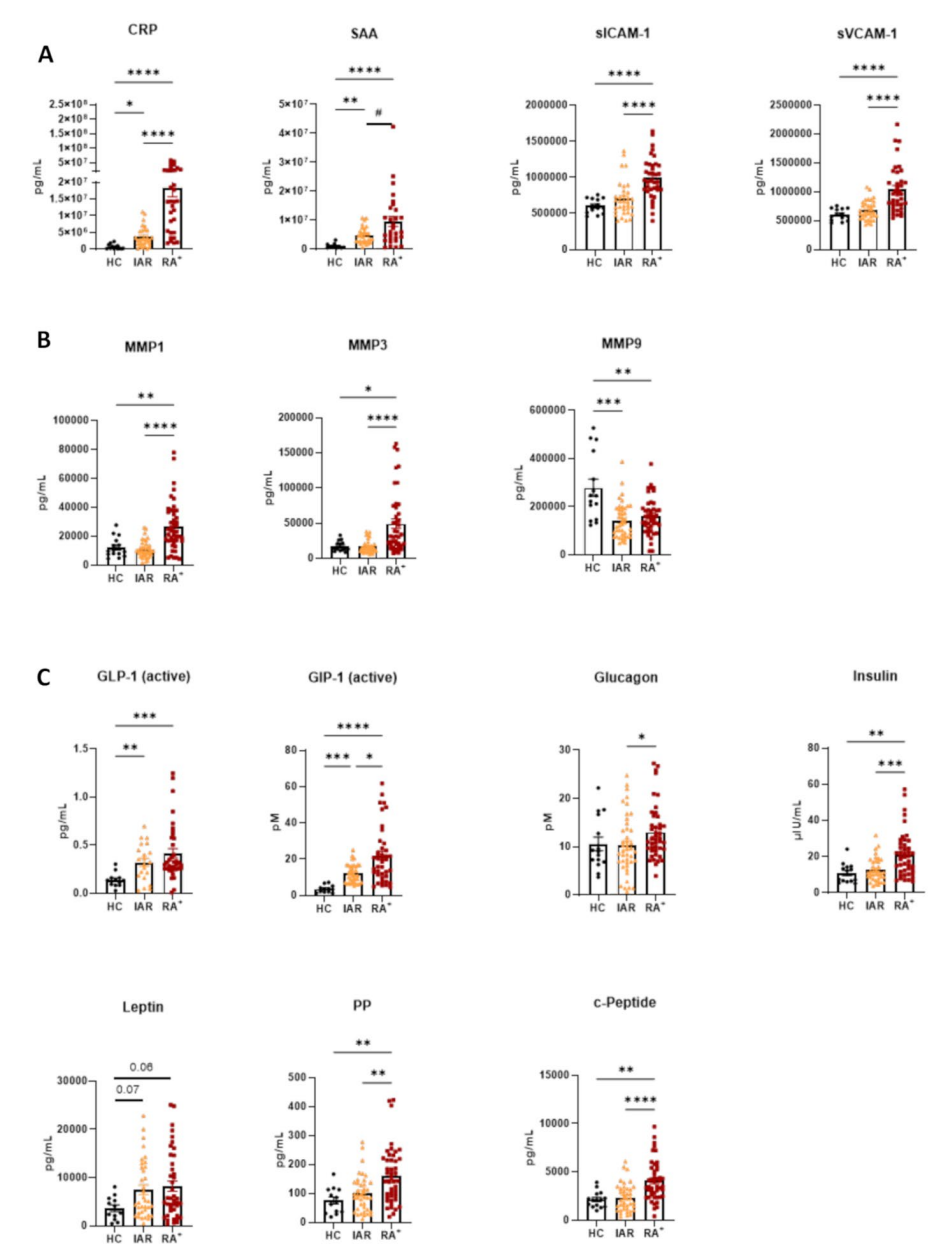
Vascular, metabolic and MMPs serum markers precede RA disease onset. Serum from HC (*n* = 15), IAR (*n* = 44) and RA^+^ (*n* = 53) patients were collected, and MSD multiplex analysis was performed. **(A)** vascular markers **(B)** MMPs, and **(C)** metabolic markers. Data are presented as Mean ± SEM and statistical differences among groups were obtained with non-parametric One-way ANOVA analysis (Kruskal-Wallis test with Dunn’s post-hoc test). *p < 0.05, ** *p* < 0.01, *** *p* < 0.001, **** *p* < 0001. Due the high heterogenicity of the samples, selective pairs were further analysed with Mann–Whitney t-test. ^#^*p* < 0.05, ^##^ *p* < 0.01, ^###^ *p* < 0.001, ^####^ *p* < 0001

Having established that selective soluble molecules are altered in the serum of RA patients specifically, we next investigated whether these markers could precede disease onset. Therefore, we measured serum markers in ‘individuals at risk’ (IAR), a cohort of individuals which have RA autoantibodies in circulation, but have not developed any clinical symptoms, however they are at risk of developing seropositive RA within their life time [30, 31]. We compared the value of serum markers in IAR vs. RA^+^ patients. Interestingly, there was a stepwise increase in inflammatory/vascular markers, CRP (*p* < 0.05) and SAA (*p* < 0.01), from HC, to IAR, to RA^+^ (Fig. 4A), suggesting that the increase in these markers is associated with susceptibility to the development of RA.

In contrast, MMP9 was significantly decreased in the serum of IAR (*p* < 0.001), at levels even lower than those observed in RA^+^(Fig. 4B).

When investigating the metabolic markers, a stepwise increase was observed for active GLP-1 (*p* < 0.01 vs. HC) and GIP-1 (*p* < 0.001 vs. HC) and Leptin (*p* = 0.07 vs. HC) (Fig. 4C), suggesting these markers were already altered in ‘individuals at risk’ of developing RA.

We next stratified the individual’s at-risk cohort into non-convertors and convertors to RA^+^, within the timeframe of the study (2 years). Interestingly, we observed that IAR convertors displayed higher levels of CRP (*p* < 0.01), SAA (*p* < 0.01), Leptin (*p* = 0.09) and PP (*p* < 0.05) (Supplementary Fig. 4), when compared to individuals that did not convert, thus displaying levels like the one observed in established seropositive RA patients. Altogether, this suggest that selective markers might be a predictor of disease conversion.

### Selective inflammatory, metabolic mediators and MMPs could be predictive of disease

**Fig. 5:**
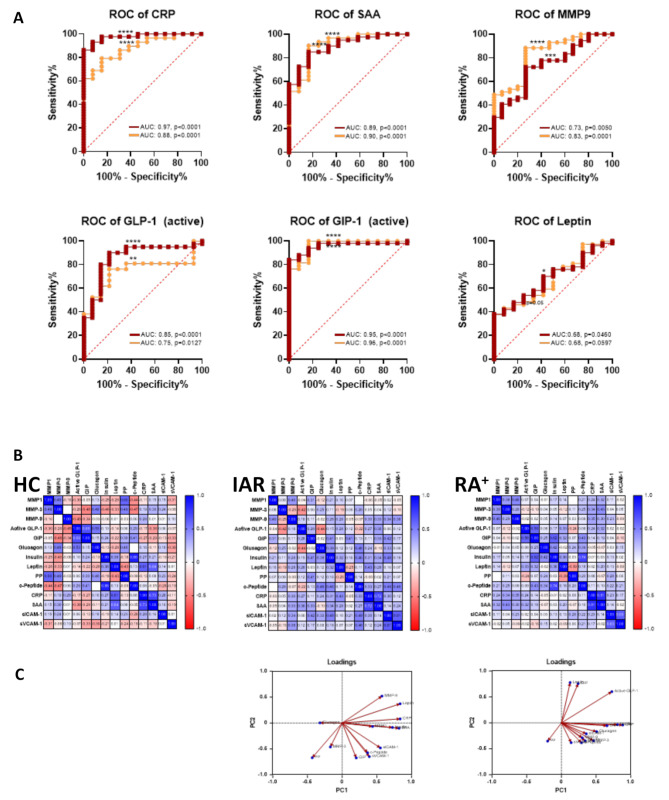
Specificity and sensitivity of serum markers in IAR and RA^+^ patients. **(A)** Receiver operating characteristic (ROC) curves for RA^+^ vs. HC (in red) and IAR vs. HC (in orange) with Wilson/Brown method and CI of 95%. AUC under the curves and p value are expressed for selective serum markers. *p < 0.05, ** *p* < 0.01, *** *p* < 0.001, **** *p* < 0001. **(B)** Non-parametric Spearman correlation matrixes were obtained and heatmaps created for HC, IAR and RA^+^ patients. p-values associated with the correlation matrix can be found in the Supplementary Fig. 5. **(C)** PCA analysis for IAR and RA^+^ patients. Standardized analysis was used for the PCA analysis and loadings for PC1 and PC2 are shown

To investigate whether these soluble markers displayed high sensitivity and could be predictive of RA onset, we compared ROC curves of RA^+^ vs. HC (Fig. 5A red), to the one obtained from IAR and HC (Fig. 5A in orange). Interestingly CRP, SAA and GIP-1 curves overlapped and displayed high sensitivity (*p* < 0.0001). In addition, MMP9 (RA^+^ *p* < 0.0001, IAR *p* < 0.001) GLP-1 (RA^+^ *p* < 0.0001, IAR *p* < 0.01), and Leptin (RA^+^ *p* < 0.05, IAR *p* = 0.06) also showed comparative profiles.

To evaluate the correlation degrees of serums markers in HC vs. IAR vs. RA, we performed correlations matrixes (Fig. 5B). The degree of correlation is displayed, and it is clearly visible that in HC there is a small of degree of correlation of these markers, including, for example MMPs correlating to each other (Supplementary Fig. 5). However, in IAR and RA^+^ comparable degrees of correlation can be observed, with IAR having correlations with HC and RA^+^. Interestingly, selective markers, including sICAM-1, displayed a higher degree of correlation with MMPs and metabolic markers in IAR in respect to RA^+^ (Supplementary Fig. 5).

PCA analysis displayed that in both IAR and RA^+^ patients, serum markers clustered in 3 areas, with Leptin being present in Cluster 1 in both cohorts For Cluster 3, SAA, sICAM-1, c-Peptide, sVCAM-1 and MMP1 clustered together in both IAR and RA^+^ (Fig. 5C), thus suggesting that the measurements of these markers together might aid in identifying individuals at risk who may convert to RA^+^.

## Discussion

Rheumatoid and Psoriatic arthritis have overlapping clinical manifestations, especially at the early phase of the disease, which render a specific diagnosis difficult [1, 32, 33]. One of the main biomarkers which can distinguish RA from PsA, are the presence/absence of autoantibodies, however a proportion of RA patients do not present with autoantibodies, and recent studies suggest that antibodies against citrullinated proteins (ACPAs) are also found in 5.0–17.5% of PsA patients [34, 35]. In light of these observations, identifying novel biomarkers which distinguish RA from PsA, could help in disease stratification, and in an early specific diagnosis.

In this work, we explore the presence of 14 serum proteins in RA and PsA patients, in the area of vascularity, metabolism and cartilage degradation, and we investigated how these could be differentially expressed between the two diseases. CRP is the gold standard biomarker in the monitoring of inflammatory diseases, and especially in RA [10], whereas in PsA, only ~ 50% of the patients have high levels of this marker, also in the presence of active disease [36]. Our results are consistent with these observations, displaying high levels of CRP in both RA and PsA, but with a significantly higher level observed in RA vs. PsA. SAA protein is expressed in inflamed synovial tissue, therefore it is considered a better marker for Inflammatory Rheumatic Diseases (IRD) [14]. Our data demonstrated that, like CRP, this marker is upregulated in RA patients at higher level in respect to PsA, thus suggesting a more significant role in RA vs. PsA diseases. Soluble ICAM-1 and VCAM-1 have been shown to be increased in the serum of RA patients and correlate with disease activity [11], however little is known about their role in PsA patients. Our data suggest that both sICAM-1 and sVCAM-1 are expressed in a RA > PsA > HC fashion, with sVCAM-1 being significantly higher in RA vs. PsA patients. Interestingly, among these markers, CRP and sICAM-1 correlated with disease activity (DAS28) in PsA at a higher degree than the one observed in RA. In addition, both sICAM-1 and sVCAM-1 were higher in the male cohort in respect to the female cohort, and sICAM-1 displayed a higher level in patients with a BMI > 25 in PsA patients.

When stratifying RA patients into seropositive and seronegative, all four vascular markers were found to be higher in the RA^+^ serum, thus suggesting an enhanced inflammatory profile in this subgroup of patients, consistent with previous studies [29, 37].

MMPs have an important role in the destruction of articular cartilage in both RA and PsA [38, 39]. In our cohort, we observed that both MMP1 and 3 were increased in the serum of RA patients compared to healthy and PsA. This is in agreement with previous studies which have shown an increase in serum MMP1 and MMP3, and their association with erosion and disease progression in RA [17, 40]. Furthermore, to the best of our knowledge, we are the first to display a difference in expression between RA and PsA. Interestingly, we observed that MMP9 was significantly downregulated in both RA and PsA patients (HC > RA > PsA). The literature on serum MMP9 levels is contradictory, with previous studies showing either an increase [41] or a decrease of MMP9 [42] in the serum of RA patients. MMP9 has been shown to be sensitive to biologics treatments, including Infliximab [43]. When stratifying our cohort into naïve, patients receiving bDMARDs or csDMARDS, we observed that patients on biologics showed the highest decrease in serum MMP9 in respect to healthy subjects (Supplementary Fig. 6), thus agreeing with previous observations. Interestingly, MMP3 showed a trending increase in the high DAS28 cohort in RA only, thus confirming it may act as a specific biomarker for RA. Interesting, MMP3 was higher in the male cohort in RA only, thus suggesting a gender confounder in its regulation.

Obesity and increased body mass index have been associated with a higher severity of RA and PsA disease [27, 44–46], however the results remain elusive and directive comparative studies are required. Here we observed that metabolic PP and c-Peptide were increased exclusively in the serum of RA patients, with levels in PsA similar to HC. GIP-1 was increased in a RA > PsA > HC fashion. Interestingly, GLP-1, Insulin and Leptin were increased in PsA more than RA and HC. Of these, Leptin was higher in high DAS28 samples in PsA, while PP was higher in the RA high DAS28 cohort and most of the metabolic markers were higher in the high BMI group, thus confirming their connection with obesity. This is the first study which directly compares serum metabolic markers between diseases and identifies specific disease signatures between RA and PsA.

ROC analysis identified specific markers highly sensitive for RA but not PsA, these included sICAM-1, MMP1, MMP3, PP and c-Peptide, therefore we believe that measuring these markers, together with the classical CRP and SAA, could aid in discriminating RA from PsA diseases, thus helping with diagnosis and specific treatment. In addition, correlation matrixes confirmed a high degree of correlation of these markers in RA, among themselves and in relation to the classical inflammatory markers, CRP and SAA, thus making them great candidates for disease stratification. A larger cohort of patients is necessary to validate these observations.

In the second part of the study, we investigated whether the specific RA serum markers could precede disease onset, and for this reason we measured serum levels in individuals-at-risk of developing RA. These individuals already present autoantibodies in circulation and have a high risk of developing RA, but they still don’t present with clinical symptoms and inflammation [8, 9]. Previous studies from our team have demonstrated that these individuals already present with cellular dysregulation, with circulatory cells being metabolically active [47, 48].

Interestingly, in our cohort, CRP, SAA, GLP-1, GIP-1, Leptin and PP were already significantly increased in the serum of IAR when compared to HC, with levels in between HC and RA (RA > IAR > HC), thus indicating that these markers could predict disease onset. Interestingly, CRP, SAA, Leptin and PP markers were predictor proteins for RA conversions, as their serum level in the IAR converted to RA, were similar to the one observed in RA^+^. These markers all showed a great degree of sensitivity and fully overlapped with RA ROC curves, and had a high degree of correlation among themselves, thus making them suitable candidate for RA disease predictors.

One of the limitations of this study, is that although we identified serum proteins which may predict disease onset, it is difficult to define whether these markers are correlative or causative. Our data shows that levels were higher in convertors vs. non-convertors at baseline, suggesting that the identified markers must have some impact on progression to disease. However, to determine if this is causative or correlative is difficult. One approach would be to perform family studies, where markers could be measured in family members who are not defined as ‘IAR’. In addition, a larger cohort of IAR convertors and non-convertors should aid in confirming some of these findings.

Overall, this study identified for the first time serum proteins sICAM-1, MMP1, MMP3, PP, c-Peptide, CRP and SAA which are specific for RA disease and could aid in discriminating RA from PsA patients, especially in the early phase of the diseases. In addition, this study identified that CRP, SAA, GLP-1, GIP-1, Leptin and PP serum proteins precede disease onset, as they are already altered in the serum of IAR. Of these, CRP, SAA, Leptin and PP may predict IAR conversion to RA^+^, thus making them suitable candidates for disease progression.

## Electronic supplementary material

Below is the link to the electronic supplementary material.

Supplementary Material 1

Supplementary Material 2

## Data Availability

No datasets were generated or analysed during the current study.
