# Supplementary material for: Investigation of serum biomarkers in rheumatoid and psoriatic arthritis patients for disease-specific signatures
Source: Arthritis Res Ther. 2025 Jul 10;27:147. doi: 10.1186/s13075-025-03608-6 (PMC12243258; doi:10.1186/s13075-025-03608-6)

Supplementary Table 1

HC	Age vs. MMP1	Age vs. MMP-3	Age vs. MMP-9	Age vs. Active GLP-1	Age vs. GIP	Age vs. Glucagon	Age vs. Insulin	Age vs. Leptin	Age vs. PP	Age vs. c-Peptide	Age vs. CRP	Age vs. SAA	Age vs. sICAM-1	Age vs. sVCAM-1
**Spearman r**														
**r**	0.3682	-0.1808	0.01985	-0.3418	0.06174	-0.3859	-0.05292	0.03087	0.05072	-0.1411	-0.07497	0.01985	0.3881	-0.1257
**95% confidence interval**	-0.2184 to 0.7594	-0.6591 to 0.4016	-0.5289 to 0.5569	-0.7463 to 0.2471	-0.4980 to 0.5851	-0.7680 to 0.1988	-0.5793 to 0.5046	-0.5209 to 0.5644	-0.5062 to 0.5778	-0.6354 to 0.4352	-0.5938 to 0.4879	-0.5289 to 0.5569	-0.1963 to 0.7690	-0.6260 to 0.4479
**P value**														
**P (two-tailed)**	0.1944	0.5332	0.9484	0.2302	0.8343	0.1726	0.8582	0.9183	0.8641	0.6281	0.7988	0.9484	0.1701	0.6666
**P value summary**	ns	ns	ns	ns	ns	ns	ns	ns	ns	ns	ns	ns	ns	ns
**RA**														
**r**	0.09367	0.112	-0.0233	0.3014	0.2672	0.05401	0.05871	0.1237	0.1469	0.1498	0.1074	0.1845	0.03823	0.1043
**95% confidence interval**	-0.1838 to 0.3573	-0.1659 to 0.3733	-0.2942 to 0.2510	0.003348 to 0.5503	-0.008751 to 0.5053	-0.2246 to 0.3245	-0.2228 to 0.3312	-0.1596 to 0.3882	-0.1366 to 0.4081	-0.1308 to 0.4083	-0.1785 to 0.3766	-0.1013 to 0.4421	-0.2449 to 0.3154	-0.1815 to 0.3739
**P value**														
**P (two-tailed)**	0.4964	0.4158	0.8659	0.0418	0.0508	0.6981	0.6763	0.3775	0.2939	0.2795	0.4484	0.1905	0.7879	0.4618
**P value summary**	ns	ns	ns	*	ns	ns	ns	ns	ns	ns	ns	ns	ns	ns
**PsA**														
**r**	0.009401	0.1034	0.02573	-0.0013	0.2276	0.01781	0.05888	0.2811	0.2954	0.004524	0.1226	0.2335	0.06952	-0.09281
**95% confidence interval**	-0.4151 to 0.4306	-0.3341 to 0.5043	-0.4015 to 0.4438	-0.4438 to 0.4417	-0.2161 to 0.5934	-0.4082 to 0.4374	-0.3733 to 0.4701	-0.1723 to 0.6362	-0.1457 to 0.6385	-0.4288 to 0.4361	-0.1343 to 0.3641	-0.02263 to 0.4608	-0.1865 to 0.3167	-0.3377 to 0.1638
**P value**														
**P (two-tailed)**	0.966	0.6387	0.9072	0.9955	0.2963	0.9357	0.7896	0.205	0.1712	0.9841	0.3344	0.0655	0.5852	0.4657
**P value summary**	ns	ns	ns	ns	ns	ns	ns	ns	ns	ns	ns	ns	ns	ns
**IAR**														
**r**	0.03734	0.2521	-0.1038	0.1959	0.4951	-0.09356	-0.01088	-0.03394	0.3492	0.08191	0.1883	0.2388	0.0943	0.05371
**95% confidence interval**	-0.2745 to 0.3420	-0.06130 to 0.5203	-0.3996 to 0.2117	-0.2585 to 0.5793	0.2201 to 0.6972	-0.3909 to 0.2215	-0.3185 to 0.2988	-0.3390 to 0.2776	0.04540 to 0.5938	-0.2365 to 0.3844	-0.1646 to 0.4985	-0.1128 to 0.5372	-0.2563 to 0.4230	-0.2940 to 0.3889
**P value**														
**P (two-tailed)**	0.8121	0.1029	0.5078	0.3822	0.0007	0.5507	0.9448	0.829	0.0217	0.6061	0.2787	0.1671	0.59	0.7593
**P value summary**	ns	ns	ns	ns	***	ns	ns	ns	*	ns	ns	ns	ns	ns

Supplementary Figure 1

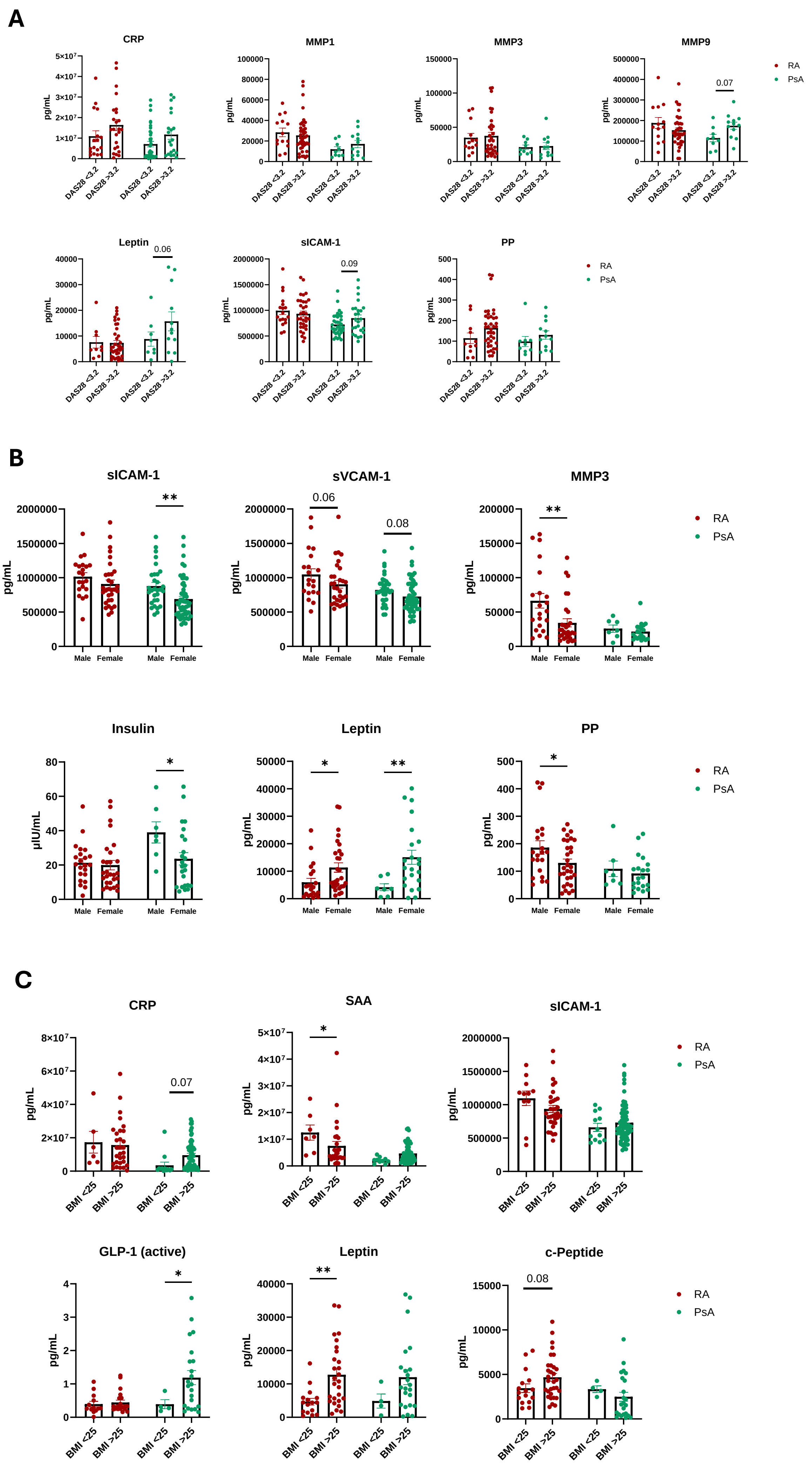

Supplementary Figure 2

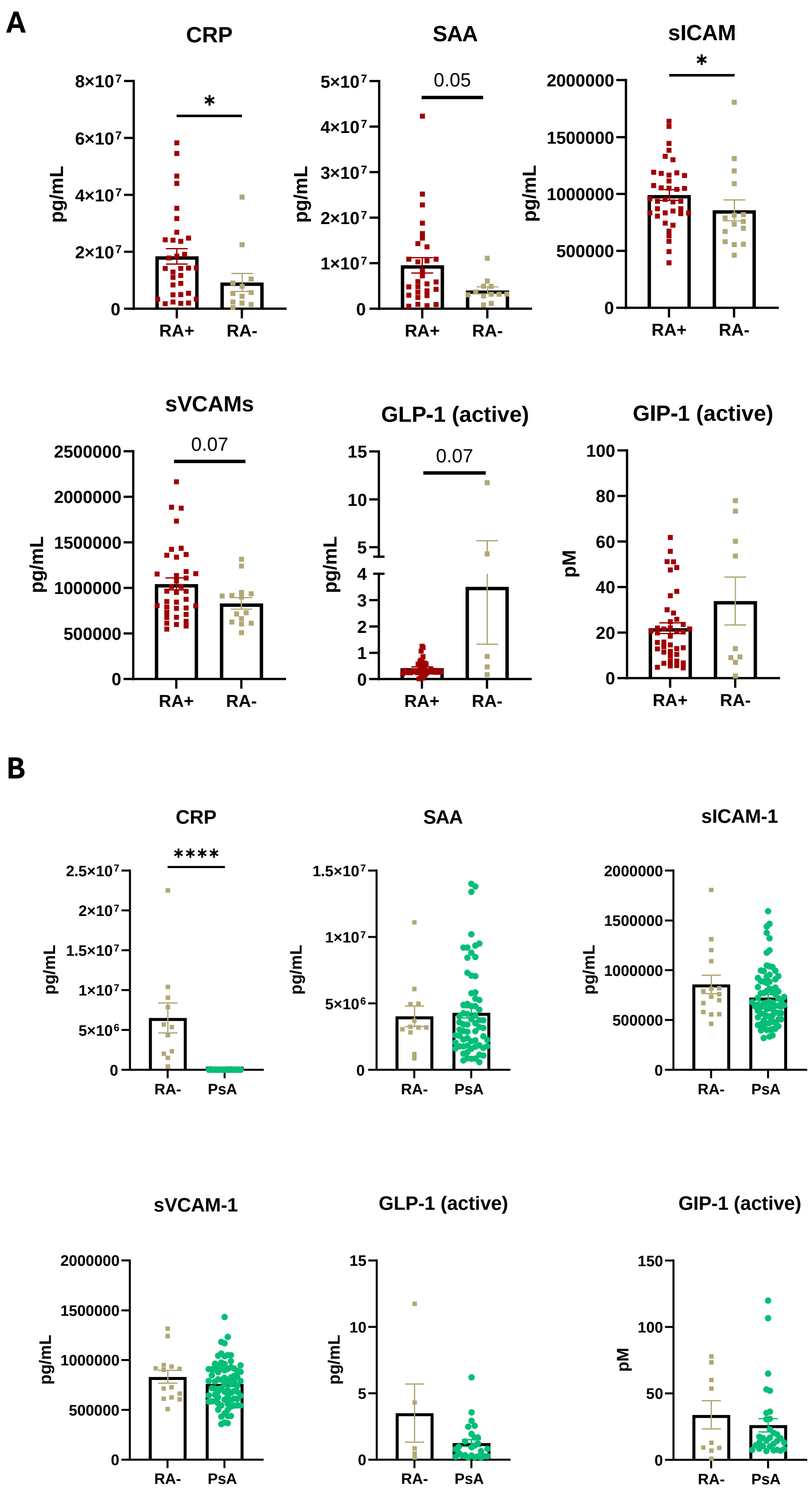

Supplementary Figure 3

A

RA	MMP1	MMP-3	MMP-9	Active GLP-1	GIP	Glucagon	Insulin	Leptin	PP	c-Peptide	CRP	SAA	sICAM-1	sVCAM-1
MMP1		0.001302	0.000283	0.69661036	0.450272476	0.378921	0.046574954	0.29127	0.509538	0.01541969	0.04321133	0.019774931	0.09016106	0.530869715
MMP-3	0.001302		0.135098	0.749894511	0.703772715	0.329547	0.484535712	0.415381	0.429725	0.05840176	0.005286169	0.001257881	0.31763077	0.370044949
MMP-9	0.000283	0.135098		0.823233033	0.742424103	0.716263	0.056263245	0.126854	0.951226	0.10093536	0.07200536	0.221278584	0.11676629	0.735322221
Active GLP-1	0.69661	0.749895	0.823233		2.34753E-05	0.473732	0.19260014	0.036634	0.582528	0.48122144	0.1269937	0.03439279	0.76235135	0.721215453
GIP	0.450272	0.703773	0.742424	2.34753E-05		0.040669	1.07446E-06	0.011432	0.447175	0.00042319	0.112195585	0.087641206	0.55870988	0.511656534
Glucagon	0.378921	0.329547	0.716263	0.473732309	0.040668819		0.003263672	0.884433	0.051858	0.00754094	0.876531799	0.895380724	0.36334908	0.16532278
Insulin	0.046575	0.484536	0.056263	0.19260014	1.07446E-06	0.003264		0.001027	0.472444	1.5684E-13	0.574124566	0.630304613	0.21112104	0.859723106
Leptin	0.29127	0.415381	0.126854	0.036634188	0.011432256	0.884433	0.001027481		0.012184	0.02565048	0.920012324	0.506803494	0.40053194	0.522081952
PP	0.509538	0.429725	0.951226	0.582527969	0.447174718	0.051858	0.472443579	0.012184		0.11936185	0.782082885	0.734870618	0.07544199	0.49674443
c-Peptide	0.01542	0.058402	0.100935	0.481221435	0.000423193	0.007541	1.5684E-13	0.02565	0.119362		0.290575838	0.407550615	0.04656982	0.178299751
CRP	0.043211	0.005286	0.072005	0.1269937	0.112195585	0.876532	0.574124566	0.920012	0.782083	0.29057584		1.11106E-14	0.00937268	0.263572147
SAA	0.019775	0.001258	0.221279	0.03439279	0.087641206	0.895381	0.630304613	0.506803	0.734871	0.40755061	1.11106E-14		0.01478815	0.58979522
sICAM-1	0.090161	0.317631	0.116766	0.762351354	0.558709882	0.363349	0.211121038	0.400532	0.075442	0.04656982	0.009372677	0.014788151		6.51416E-08
sVCAM-1	0.53087	0.370045	0.735322	0.721215453	0.511656534	0.165323	0.859723106	0.522082	0.496744	0.17829975	0.263572147	0.58979522	6.5142E-08	

B

PsA	MMP1	MMP-3	MMP-9	Active GLP-1	GIP	Glucagon	Insulin	Leptin	PP	c-Peptide	CRP	SAA	sICAM-1	sVCAM-1
MMP1		0.703456	**0.099907**	0.489866943	0.218060074	**0.087641**	0.014900006	0.758328	0.265488	0.32589863	0.594405816	0.939602142	0.01707964	0.023563691
MMP-3	0.703456		0.224791	0.105347723	**0.058360629**	0.788364	0.015801476	0.933206	0.194527	0.74793345	0.108119962	**0.077917104**	0.64847802	0.867594943
MMP-9	0.099907	0.224791		0.450325442	0.906584492	0.114802	0.594168258	**0.064623**	0.135997	0.56941869	0.155310304	0.203140129	0.91554088	0.927566286
Active GLP-1	0.489867	0.105348	0.450325		0.168577043	0.040458	0.28851259	0.613418	**0.096539**	0.19042	0.764664313	0.470298436	0.76466431	0.963939964
GIP	0.21806	**0.058361**	0.906584	0.168577043		0.995542	0.007621096	0.741311	0.280896	0.19060939	0.552569364	0.532141654	0.42652293	0.132381469
Glucagon	**0.087641**	0.788364	0.114802	0.040457634	0.995542285		0.350949549	0.594168	0.330388	0.00158933	0.165190993	0.317591121	0.48270901	0.987912067
Insulin	0.0149	0.015801	0.594168	0.28851259	0.007621096	0.35095		0.814343	0.582479	0.02451927	0.463581475	0.444846111	0.37401018	0.043844666
Leptin	0.758328	0.933206	**0.064623**	0.613417665	0.741311377	0.594168	0.814342822		0.633823	0.25113145	0.100786489	0.738509838	0.9035096	0.808321229
PP	0.265488	0.194527	0.135997	**0.096538578**	0.280895671	0.330388	0.582478861	0.633823		0.73360763	0.715635727	0.374010185	0.84378111	0.512102399
c-Peptide	0.325899	0.747933	0.569419	0.190419995	0.190609387	0.001589	0.024519268	0.251131	0.733608		0.903509596	0.552569364	0.77320805	0.056695961
CRP	0.594406	0.10812	0.15531	0.764664313	0.552569364	0.165191	0.463581475	0.100786	0.715636	0.9035096		1.30193E-10	0.00049202	0.004856491
SAA	0.939602	**0.077917**	0.20314	0.470298436	0.532141654	0.317591	0.444846111	0.73851	0.37401	0.55256936	1.30193E-10		0.06666638	0.305792072
sICAM-1	0.01708	0.648478	0.915541	0.764664313	0.426522931	0.482709	0.374010185	0.90351	0.843781	0.77320805	0.000492021	**0.066666377**		1.95741E-08
sVCAM-1	0.023564	0.867595	0.927566	0.963939964	0.132381469	0.987912	0.043844666	0.808321	0.512102	**0.05669596**	0.004856491	0.305792072	1.9574E-08	

Supplementary Figure 4

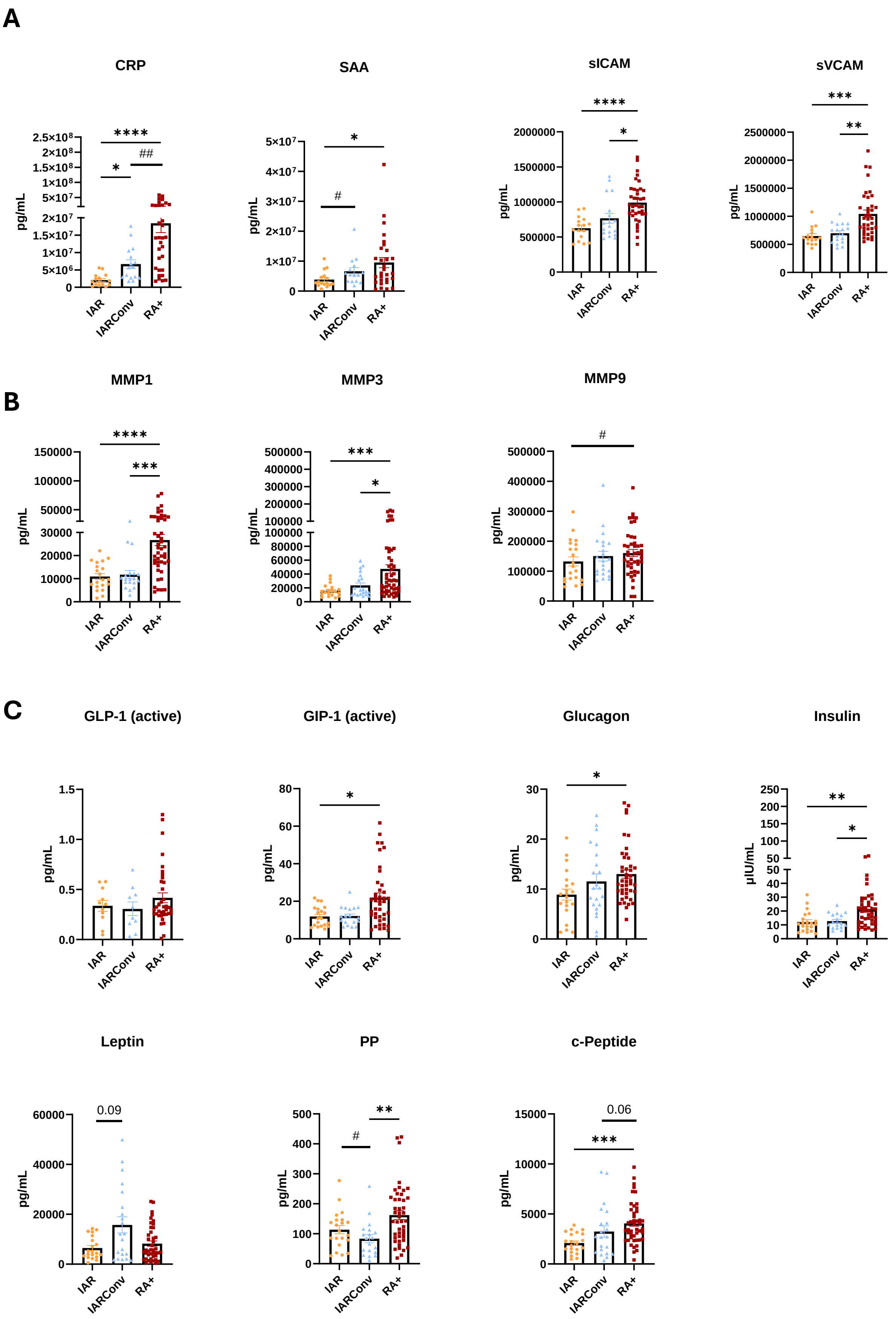

Supplementary Figure 5

A

HC	MMP1	MMP-3	MMP-9	Active GLP-1	GIP-1	Glucagon	Insulin	Leptin	PP	c-Peptide	CRP	SAA	sICAM-1	sVCAM-1
MMP1		0.06441	0.55798	0.27673607	0.86261	0.64823	0.37483	0.32029	0.02035	0.10631	0.71564	0.6158	0.6158	0.27354
MMP-3	0.06441		0.99485	0.3538308	0.07347	0.06441	0.14462	0.23168	0.06883	0.07834	0.5121	0.29508	0.39979	0.79655
MMP-9	0.55798	0.99485		0.09437334	0.22121	0.93357	0.83239	0.96425	0.92345	0.82245	0.33317	0.97582	0.97582	0.72704
Active GLP-1	0.27674	0.35383	0.09437		0.00608	0.04375	0.73371	0.62974	0.84248	0.28879	0.5629	0.18623	0.83192	0.8201
GIP	0.86261	0.07347	0.22121	0.00607596		0.60236	0.38923	0.43425	0.27674	0.12618	0.34917	0.30998	0.64848	0.25302
Glucagon	0.64823	0.06441	0.93357	0.04375223	0.60236		0.6389	0.32029	0.14462	0.99485	0.57332	0.70428	0.53214	0.20314
Insulin	0.37483	0.14462	0.83239	0.73371415	0.38923	0.6389		0.16915	0.51505	3.5E-05	0.34917	0.46358	0.73851	0.47309
Leptin	0.32029	0.23168	0.96425	0.62974102	0.43425	0.32029	0.16915		0.11592	0.6389	0.02237	0.01708	0.62662	0.97582
PP	0.02035	0.06883	0.92345	0.84248222	0.27674	0.14462	0.51505	0.11592		0.74348	0.67061	0.79655	0.43563	0.41749
c-Peptide	0.10631	0.07834	0.82245	0.28878713	0.12618	0.99485	3.5E-05	0.6389	0.74348		0.79655	0.87955	0.33317	0.52208
CRP	0.71564	0.5121	0.33317	0.56290338	0.34917	0.57332	0.34917	0.02237	0.67061	0.79655		0.00401	0.33317	0.70428
SAA	0.6158	0.29508	0.97582	0.18623043	0.30998	0.70428	0.46358	0.01708	0.79655	0.87955	0.00401		0.57332	0.5121
sICAM-1	0.6158	0.39979	0.97582	0.83192085	0.64848	0.53214	0.73851	0.62662	0.43563	0.33317	0.33317	0.57332		0.98791
sVCAM-1	0.27354	0.79655	0.72704	0.82010364	0.25302	0.20314	0.47309	0.97582	0.41749	0.52208	0.70428	0.5121	0.98791	

B

IAR	MMP1	MMP-3	MMP-9	Active GLP-1	GIP-1	Glucagon	Insulin	Leptin	PP	c-Peptide	CRP	SAA	sICAM-1	sVCAM-1
MMP1		0.65237	0.01105	0.86410752	0.06898	0.44944	0.23375	0.33117	0.66852	0.9922	0.76458	0.80416	0.9145	0.81268
MMP-3	0.65237		0.12354	0.0913115	0.73718	0.12167	0.5428	0.25572	0.63731	0.0848	0.80164	0.82925	0.53525	0.57753
MMP-9	0.01105	0.12354		0.44715972	0.52285	0.89752	0.09205	0.08172	0.75537	0.6371	0.10876	0.07293	0.04786	0.02737
Active GLP-1	0.86411	0.09131	0.44716		0.26843	0.04797	0.5479	0.22956	0.62238	0.32751	0.37341	0.22401	0.83076	0.66446
GIP	0.06898	0.73718	0.52285	0.26842513		0.9257	0.01478	0.84049	0.0111	0.44669	0.58542	0.0924	0.02316	0.37992
Glucagon	0.44944	0.12167	0.89752	0.04796936	0.9257		0.01684	0.47036	0.47474	0.00147	0.20309	0.6093	0.05649	0.62612
Insulin	0.23375	0.5428	0.09205	0.54790067	0.01478	0.01684		0.06091	0.35138	0.00457	0.10799	0.08038	0.00671	0.34546
Leptin	0.33117	0.25572	0.08172	0.22955839	0.84049	0.47036	0.06091		0.13089	0.5072	0.02156	0.14862	0.57887	0.70931
PP	0.66852	0.63731	0.75537	0.62237898	0.0111	0.47474	0.35138	0.13089		0.40615	0.78206	0.91919	0.25234	0.71226
c-Peptide	0.9922	0.0848	0.6371	0.32751027	0.44669	0.00147	0.00457	0.5072	0.40615		0.08476	0.16147	0.00357	0.00495
CRP	0.76458	0.80164	0.10876	0.37340809	0.58542	0.20309	0.10799	0.02156	0.78206	0.08476		9.2E-06	0.10955	0.53098
SAA	0.80416	0.82925	0.07293	0.22401362	0.0924	0.6093	0.08038	0.14862	0.91919	0.16147	9.2E-06		0.46726	0.20131
sICAM-1	0.9145	0.53525	0.04786	0.83075515	0.02316	0.05649	0.00671	0.57887	0.25234	0.00357	0.10955	0.46726		0.00012
sVCAM-1	0.81268	0.57753	0.02737	0.66445685	0.37992	0.62612	0.34546	0.70931	0.71226	0.00495	0.53098	0.20131	0.00012	

C

RA^{+}	MMP1	MMP-3	MMP-9	Active GLP-1	GIP-1	Glucagon	Insulin	Leptin	PP	c-Peptide	CRP	SAA	sICAM-1	sVCAM-1
MMP1		0.00802	0.0009	0.66321773	0.77049	0.96093	0.25062	0.34388	0.9815	0.15986	0.17177	0.06855	0.54313	0.92825
MMP-3	0.00802		0.04288	0.4649117	0.5085	0.77167	0.75244	0.43876	0.47705	0.09691	0.04578	0.01056	0.84292	0.77506
MMP-9	0.0009	0.04288		0.87693631	0.90788	0.76311	0.36447	0.66512	0.82134	0.54398	0.04547	0.0865	0.18243	0.62004
Active GLP-1	0.66322	0.46491	0.87694		5.2E-05	0.62217	0.30105	0.03721	0.12685	0.55618	0.18592	0.07278	0.90963	0.92385
GIP	0.77049	0.5085	0.90788	5.1733E-05		0.09682	5.4E-06	0.03056	0.6334	0.00281	0.44416	0.29346	0.82894	0.37958
Glucagon	0.96093	0.77167	0.76311	0.62217342	0.09682		0.00351	0.88774	0.11113	0.01992	0.79879	0.69447	0.87601	0.39712
Insulin	0.25062	0.75244	0.36447	0.30105368	5.4E-06	0.00351		0.01584	0.86017	2.9E-09	0.96426	0.85774	0.46907	0.92227
Leptin	0.34388	0.43876	0.66512	0.03721367	0.03056	0.88774	0.01584		0.00766	0.22705	0.89542	0.26496	0.48658	0.62639
PP	0.9815	0.47705	0.82134	0.12685158	0.6334	0.11113	0.86017	0.00766		0.18542	0.64708	0.71229	0.12727	0.74228
c-Peptide	0.15986	0.09691	0.54398	0.55617919	0.00281	0.01992	2.9E-09	0.22705	0.18542		0.56042	0.57623	0.21807	0.238
CRP	0.17177	0.04578	0.04547	0.18592482	0.44416	0.79879	0.96426	0.89542	0.64708	0.56042		4E-10	0.15527	0.30626
SAA	0.06855	0.01056	0.0865	0.07278351	0.29346	0.69447	0.85774	0.26496	0.71229	0.57623	4E-10		0.40823	0.91477
sICAM-1	0.54313	0.84292	0.18243	0.90962912	0.82894	0.87601	0.46907	0.48658	0.12727	0.21807	0.15527	0.40823		1.9E-05
sVCAM-1	0.92825	0.77506	0.62004	0.92385315	0.37958	0.39712	0.92227	0.62639	0.74228	0.238	0.30626	0.91477	1.9E-05	

Supplementary Figure 6

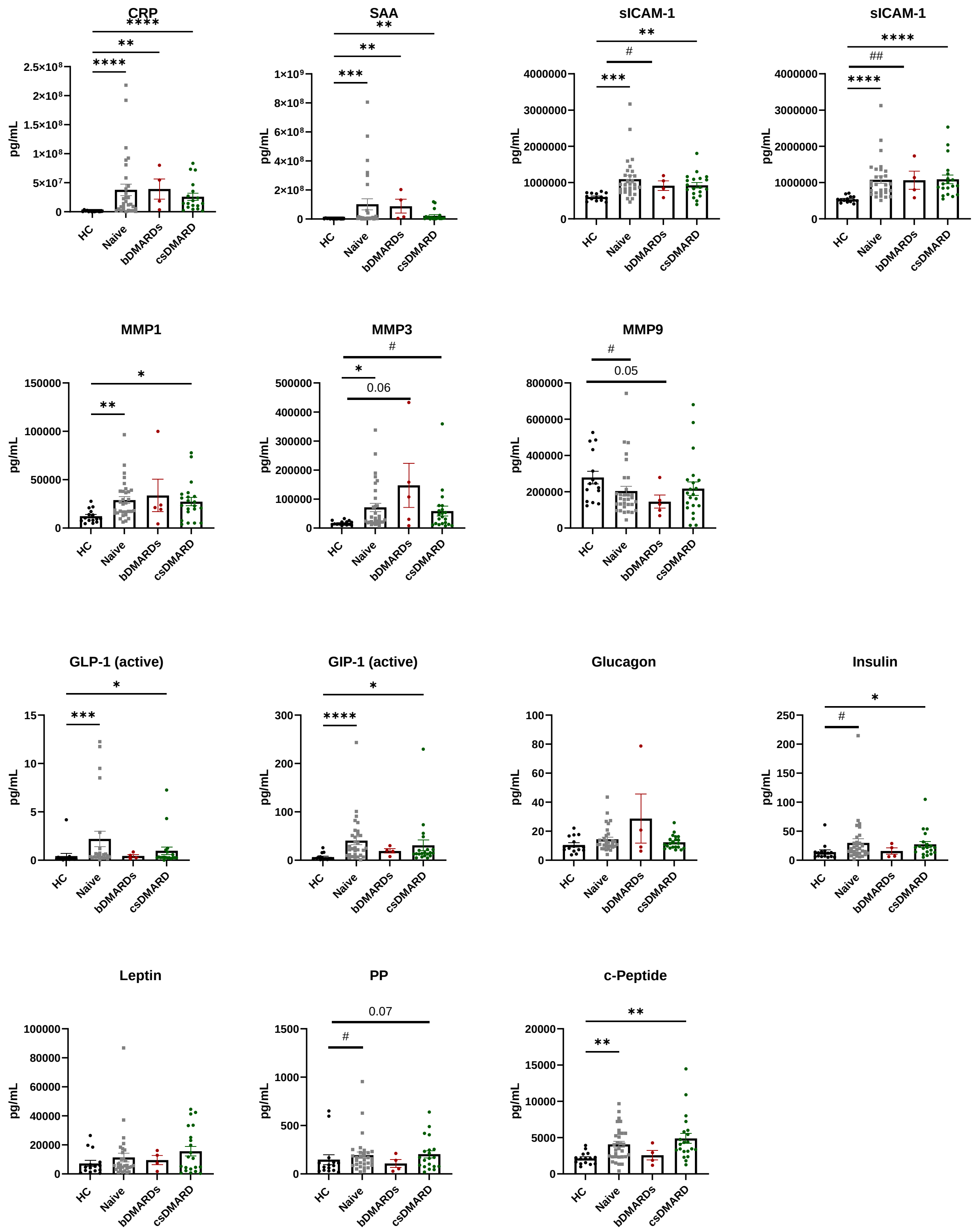

Supplement: Supplementary file 1 — Supplementary Material 1 [file 13075_2025_3608_MOESM1_ESM.pdf]
