# Supplementary material for: Investigation of serum biomarkers in rheumatoid and psoriatic arthritis patients for disease-specific signatures
Source: Arthritis Res Ther. 2025 Jul 10;27:147. doi: 10.1186/s13075-025-03608-6 (PMC12243258; doi:10.1186/s13075-025-03608-6)
Supplement: Supplementary file 2 — Supplementary Material 2 [file 13075_2025_3608_MOESM2_ESM.docx]

**Supplementary Table 1. Correlation of vascular, metabolic and MMPs serum markers with age.** Serum from HC (n=15), IAR (n=44), RA (n=74) and PsA (n=97) patients were collected, and MSD multiplex analysis was performed for vascular markers, MMPs, and metabolic markers. Correlation of serum markers with age were assessed with Non-parametric Spearman correlation with 95% confidence interval.

**Supplementary Figure 1. Vascular, metabolic and MMPs serum markers stratified by disease activity, sex and BMI**. Serum from RA (n=74) and PsA (n=97) patients were collected, and MSD multiplex analysis was performed for vascular markers, MMPs, and metabolic markers. Stratification of patients based on **A)** DAS28 activity (cut off 3.2), **B)** sex, and **C)** BMI (cut off 25). Data are presented as Mean ± SEM and statistical differences among groups were obtained with non-parametric One-way ANOVA analysis (Kruskal-Wallis test with Dunn’s post-hoc test). *p <0.05, ** p <0.01, *** p <0.001, **** p <0001. Due the high heterogenicity of the samples, selective pairs were further analysed with Mann–Whitney t-test. ^#^p <0.05, ^##^ p <0.01, ^###^ p <0.001, ^####^ p <0001.

**Supplementary Figure 2. Vascular, metabolic and MMPs serum markers stratified by RA seropositive and seronegative status**. Serum from RA (n=74) patients were collected, and MSD multiplex analysis was performed for vascular markers, MMPs, and metabolic markers. **A)** Patients were stratified by the presence/absence of autoantibodies (RF and ACPA), and differences between RA^-^ and RA^+^ are shown for selected markers. **B)** RA^-^ *vs* PsA patient data are represented. Data are presented as Mean ± SEM and statistical differences among groups were obtained with non-parametric One-way ANOVA analysis (Kruskal-Wallis test with Dunn’s post-hoc test). *p <0.05, ** p <0.01, *** p <0.001, **** p <0001. Due the high heterogenicity of the samples, selective pairs were further analysed with Mann–Whitney t-test. ^#^p <0.05, ^##^ p <0.01, ^###^ p <0.001, ^####^ p <0001.

**Supplementary Figure 3. Selective serum markers are differentially correlated and clustered in RA and PsA patients.** Serum from RA (n=74) and PsA (n=97) patients were collected, and MSD multiplex analysis was performed. Non-parametric Spearman correlation matrixes were obtained and heatmaps created in Figure 2. p-values associated with the correlation matrix are displayed for **A)** RA and **B)** PsA patients. In red are highlighted significant correlation (p<0.05), and in orange trending significant correlations (p =0.05 – p =0.09).

**Supplementary Figure 4. Vascular, metabolic and MMPs serum markers precede RA disease onset**. Serum from HC (n=15), IAR (n=44) and RA^+^ (n=53) patients were collected, and MSD multiplex analysis was performed. IAR patients were divided into convertors (IARConv) and non-convertors, based on their conversion to RA^+^ during the timeframe of the study. **A)** vascular markers **B)** MMPs, and **C)** metabolic markers. Data are presented as Mean ± SEM and statistical differences among groups were obtained with non-parametric One-way ANOVA analysis (Kruskal-Wallis test with Dunn’s post-hoc test). *p <0.05, ** p <0.01, *** p <0.001, **** p <0001. Due the high heterogenicity of the samples, selective pairs were further analysed with Mann–Whitney t-test. ^#^p <0.05, ^##^ p <0.01, ^###^ p <0.001, ^####^ p <0001.

**Supplementary Figure 5. Selective serum marker correlation in HC, IAR and RA^+^ patients.** Serum from HC (n=15), RA^+^ (n=53) and PsA (n=97) patients were collected, and MSD multiplex analysis was performed. Non-parametric Spearman correlation matrixes were obtained and heatmaps created in Figure 5. p-values associated with the correlation matrix are displayed for **A)** HC and **B)** IAR and **C)** RA^+^ patients. In red are highlighted significant correlations (p <0.05), and in orange trending significant correlations (p =0.05 – p =0.09).

**Supplementary Figure 6. RA patient stratification based on therapy**. Serum protein levels for HC (n=15) and RA (n=73) patients are displayed. RA patients were stratified into naïve, patients, receiving bDMARDs and csDMARDs. Data are presented as Mean ± SEM and statistical differences among groups were obtained with non-parametric One-way ANOVA analysis (Kruskal-Wallis test with Dunn’s post-hoc test). *p <0.05, ** p <0.01, *** p <0.001, **** p <0001. Due the high heterogenicity of the samples, selective pairs were further analysed with Mann–Whitney t-test. ^#^p <0.05, ^##^ p <0.01, ^###^ p <0.001, ^####^ p <0001.
